# Rhizosphere Microbial Community and Metagenomic Annotation Responses in a *Vallisneria natans*–Sediment Microcosm Exposed to Trifluenfuronate and Fluopyram

**DOI:** 10.3390/microorganisms14051166

**Published:** 2026-05-21

**Authors:** Shiqi Zhang, Guo Li, Ensheng Zhu, Yu Zhao, Xiaoying Yang, Suzhen Huang, Zheng Zheng

**Affiliations:** 1 Department of Environmental Science and Engineering, Fudan University, Shanghai 200438, China; 2 College of Biological and Environmental Engineering, Zhejiang Shuren University, Hangzhou 310015, China

**Keywords:** aquatic pesticide exposure, microcosm ecotoxicology, rhizosphere microorganisms

## Abstract

Rhizosphere microorganisms play central roles in nutrient cycling and contaminant transformation in sediment-associated freshwater systems, yet their responses to newer pesticides remain insufficiently characterized. In this study, a 28-day *Vallisneria natans*–rhizosphere sediment microcosm was used to compare the effects of trifluenfuronate and fluopyram at nominal concentrations of 0.01, 0.1, and 1 mg L^−1^. Bacterial community composition was assessed using 16S rRNA gene sequencing, and shotgun metagenomic data were used to evaluate relative functional annotation patterns. Plant physiological traits and rhizosphere sediment enzyme activities were measured as ecological context for interpreting microorganism-associated responses. Fluopyram, particularly at 1 mg L^−1^, produced clearer ordination-level shifts in rhizosphere bacterial community composition than trifluenfuronate, although pairwise treatment separation was not statistically resolved after multiple-testing correction. Annotation-based metagenomic profiles also differed between the two pesticides: stronger exposure was associated with reduced relative signals for several xenobiotic-, transport-, and regulation-related annotations, while high-dose fluopyram showed a methane-metabolism-related annotation signal and high-dose trifluenfuronate showed relative enrichment of secondary-metabolism-related annotations. These microbial and annotation-profile responses coincided with stronger inhibition of *V. natans* growth and greater suppression of rhizosphere sediment enzyme activities under fluopyram exposure. Overall, fluopyram induced more consistent microorganism-associated response patterns than trifluenfuronate in the tested rooted macrophyte–sediment microcosm. The results highlight the sensitivity of rhizosphere microbial communities and metagenomic annotation profiles to pesticide exposure in sediment-associated freshwater systems.

## 1. Introduction

Sediment-associated freshwater systems host diverse microbial communities that regulate nutrient cycling and contaminant transformation. Pesticides released from agricultural activities can enter freshwater systems through runoff and leaching, and sediment sorption may prolong microbial exposure by retaining pesticide residues [1]. Therefore, pesticide inputs can modify not only exposure conditions but also sediment- and rhizosphere-associated microbial community structure and potential functional profiles.

Rooted submerged macrophytes provide a useful interface for examining pesticide effects on rhizosphere microorganisms because plant roots shape sediment microhabitats and carbon inputs, and microbial community assembly [2,3]. *Vallisneria natans*, a common rooted submerged macrophyte in shallow freshwater ecosystems, was used here as a model host to establish a plant–sediment rhizosphere microcosm [2]. In this study, plant physiological traits and rhizosphere sediment enzyme activities were included to describe the host–sediment context of microbial responses, rather than to evaluate plant toxicity as the primary endpoint. Although previous studies have addressed pesticide effects on aquatic plants or microbial community composition, comparative assessments focusing on rhizosphere bacterial community patterns together with shotgun metagenomic annotation profiles in rooted macrophyte–sediment systems remain limited [1,4].

In this study, we used a 28-day *V. natans*-rhizosphere sediment microcosm to compare microorganism-associated responses to trifluenfuronate and fluopyram under a common nominal concentration gradient. The low and medium concentrations were selected as the literature-informed nominal test levels based on reported aquatic occurrence information for fluopyram and other agricultural pesticides, whereas compound-specific environmental monitoring data for trifluenfuronate remain limited because it is a recently developed compound [5]. Therefore, the same nominal concentration gradient was used for both pesticides to enable a controlled mass-based comparison, and 1 mg L^−1^ was included as a high-end single-pulse runoff/stress scenario derived from an agricultural ditch/runoff exposure calculation and was used to compare system-level responses under stronger chemical pressure. Fluopyram is a succinate dehydrogenase inhibitor (SDHI) [6]. Trifluenfuronate is a novel mesoionic insecticide recently developed in China, for which aquatic ecotoxicological information remains limited [7]. The objectives were to characterize rhizosphere bacterial community structure and annotation-based functional profiles, together with sediment enzyme activities and plant physiological indicators as ecological context, and to examine whether microbe-associated responses differentiated fluopyram from trifluenfuronate under the tested microcosm conditions.

## 2. Materials and Methods

### 2.1. Microcosm Setup and Sample Collection

*V. natans* plants were obtained from a horticultural company in Nanjing, China, and cleaned and pre-cultured for one week in controlled laboratory conditions before microcosm construction [8]. Surface water and sediment were collected from Rihu Lake, Fudan University, Shanghai, China. Sediment samples were homogenized and sieved before use. Basic water and sediment properties are provided in Table S1.

Each 5 L microcosm contained 1.5 kg of pre-treated sediment, 4 L of lake water, and three to four pre-cultured *V. natans* individuals. Seven treatment groups were established: a solvent control (CT), three trifluenfuronate treatments (T-L, T-M, and T-H), and three fluopyram treatments (F-L, F-M, and F-H). Nominal concentrations were 10, 100, and 1000 μg L^−1^ for each pesticide. Analytical standards of trifluenfuronate and fluopyram were obtained from Shandong United Pesticide Industry (Jinan, China) and Nanjing Agrochemical Co., Ltd. (Nanjing, China), with purities of 98.5% and 96%, respectively. Stock solutions were prepared in acetone (Sinopharm Chemical Reagent Co., Ltd., Shanghai, China) and stored at 4 °C in the dark until use. Working solutions were added to the overlying water, and the final acetone concentration was kept below 0.005% (*v*/*v*) in all treatments; CT received the same acetone volume.

The experiment was conducted as a 28-day single-pulse exposure, and the initial overlying-water pesticide concentrations were verified immediately after dosing. The same nominal concentration gradient was applied to both pesticides to enable a controlled mass-based comparison. The concentration rationale, including published pesticide occurrence/risk information in Chinese surface waters and a conservative agricultural runoff/stress scenario, is provided in Supplementary Text S1 [9,10]. The initial overlying-water concentrations were 88–99% of the nominal levels. Detailed HPLC-MS/MS procedures and validation results are provided in Supplementary Texts S2 and S3 and Tables S2–S4.

Each treatment consisted of three independent microcosms (*n* = 3), and each container was treated as one biological replicate. After 28 days, rhizosphere sediment tightly associated with the roots was collected from each microcosm for microbial, metagenomic, and enzyme analyses. Plant growth and tissue samples were collected as host-context endpoints. Measurements from plants within the same container were averaged to obtain one value per microcosm, thereby avoiding pseudoreplication.

### 2.2. DNA Extraction, Sequencing, and Metagenomic Annotation

Total DNA was extracted from rhizosphere sediment using the Mag-Bind^®^ Soil DNA Kit (Omega Bio-tek, Norcross, GA, USA) according to the manufacturer’s protocol. DNA integrity was assessed by 1% agarose gel electrophoresis, and DNA concentration and purity were determined using a NanoDrop 2000 spectrophotometer (Thermo Fisher Scientific, Waltham, MA, USA).

For bacterial community profiling, the V3–V4 region of the 16S rRNA gene was amplified using primers 338F/806R [11] and sequenced on an Illumina MiSeq PE300 platform (Illumina Inc., San Diego, CA, USA). One rhizosphere sediment sample from each independent microcosm was analyzed, resulting in three biological replicates per treatment (*n* = 3). Raw 16S rRNA gene sequence data have been deposited in the NCBI database under accession number PRJNA1422131.

For shotgun metagenomic analysis, rhizosphere sediment samples from all treatments were sequenced, with three independent biological replicates per treatment (*n* = 3). Metagenomic libraries were constructed from extracted DNA fragmented to approximately 350 bp and sequenced on an Illumina HiSeq X Ten platform (Illumina Inc., San Diego, CA, USA) at Majorbio Bio-Pharm Technology Co., Ltd. (Shanghai, China). Raw metagenomic reads were quality-filtered using fastp v0.20.0. Clean reads were assembled using MEGAHIT, and contigs longer than 300 bp were retained for downstream analyses [12]. Open reading frames were predicted from assembled contigs, and a nonredundant gene catalog was generated by clustering predicted genes using CD-HIT v4.7 [13]. Functional annotation and downstream bioinformatics analyses were performed on the Majorbio Cloud Platform (Majorbio Bio-Pharm Technology Co., Ltd., Shanghai, China; accessed on 10 March 2026) [14]. Metagenomic outputs were interpreted as relative functional annotation patterns, rather than as direct measurements of realized metabolic rates or pathway activity.

### 2.3. Contextual Plant and Rhizosphere Enzyme Measurements

Plant fresh weight, leaf length, root length, and chlorophyll contents were measured after 28 days to describe host-plant status in the microcosms. Chlorophyll a and b were determined by ethanol extraction; detailed equations and pathlength correction are provided in Text S5.

Root oxidative-response indicators, including MDA, SOD, POD, and CAT, were measured as host-context variables using commercial assay kits (Nanjing Jiancheng Bioengineering Institute, Nanjing, China). Rhizosphere sediment urease, dehydrogenase, neutral phosphatase, and sucrase activities were quantified using commercial assay kits (Nanjing Jiancheng Bioengineering Institute, Nanjing, China) as integrated indicators of nitrogen transformation, microbial redox activity, phosphorus mineralization, and labile-carbon turnover, respectively. Sample pretreatment and kit information are provided in Text S5.

### 2.4. Statistical Analysis

Statistical analyses were conducted at the microcosm level with three independent biological replicates per treatment. Data are presented as mean ± standard deviation (SD). Plant and enzyme endpoints were analyzed using IBM SPSS Statistics for Windows, Version 20.0 (IBM Corp., Armonk, NY, USA), and figures were prepared using Origin 2026 (OriginLab Corporation, Northampton, MA, USA). Microbial community, LEfSe, Spearman correlation, and db-RDA analyses were performed on the Majorbio Cloud Platform (Majorbio Bio-Pharm Technology Co., Ltd., Shanghai, China; accessed on 10 March 2026). Given the limited biological replication, omics-related and multivariate results were interpreted conservatively as treatment-associated community or annotation patterns.

Plant and enzyme endpoints were analyzed by one-way ANOVA followed by Tukey’s test when assumptions of normality and variance homogeneity were met. These endpoints were interpreted together with effect direction and consistency, rather than as primary evidence for microbial mechanisms.

For microbial community analyses, Bray–Curtis distances were used for principal coordinates analysis (PCoA). Differences in community composition among treatments were assessed using permutational multivariate analysis of variance (PERMANOVA) with 999 permutations [15]. Pairwise PERMANOVA comparisons were further conducted to identify treatment-level differences, and *p* values were adjusted using the Benjamini–Hochberg procedure. Homogeneity of multivariate dispersion was additionally evaluated to assess whether significant PERMANOVA results could be influenced by differences in within-group dispersion [16].

16S rRNA gene libraries were rarefied to 28,669 valid sequences per sample and rarefaction curves approached a plateau (Supplementary Text S4 and Figure S1). The rarefaction-based normalization strategy was retained after reassessment because the microbial analyses focused mainly on diversity and Bray–Curtis-based community composition patterns rather than confirmatory differential-abundance testing.

LEfSe was used only as an exploratory screening approach, and genus-level results were further checked by Kruskal–Wallis tests with Benjamini–Hochberg correction [17]. Spearman correlation and db-RDA analyses were used to explore associations among microbial taxa, annotation profiles, pesticide gradients, and contextual biochemical variables [18]. These association analyses were not interpreted as evidence of direct causality.

## 3. Results

### 3.1. Host–Sediment Contextual Responses

Plant physiological traits and rhizosphere sediment enzyme activities were measured as host–sediment contextual endpoints for interpreting microorganism-associated responses.

**Figure 1:**
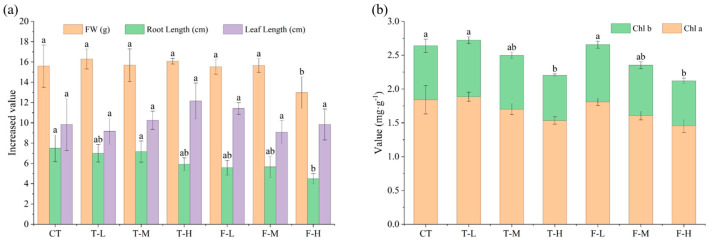
Increments in fresh weight, leaf length and root length of *V. natans* after 28 days (**a**), and changes in chlorophyll contents (**b**). Data are shown as mean ± SD (*n* = 3). Different letters indicate significant differences among treatments at *p* < 0.05.

Plant growth responses were generally limited, except under high-dose fluopyram exposure (Figure 1a). Leaf length increment did not differ significantly among treatments (*p* > 0.05), whereas F-H reduced fresh-weight gain and root elongation by 35.63% and 30.77%, respectively (*p* < 0.05). Chlorophyll responses were mainly observed at the high dose (Figure 1b). Both pesticides decreased pigment contents at 1 mg L^−1^, with Chl a and Chl b declining by 16.64% and 13.58% in T-H and by 20.93% and 16.66% in F-H, respectively.

**Figure 2:**
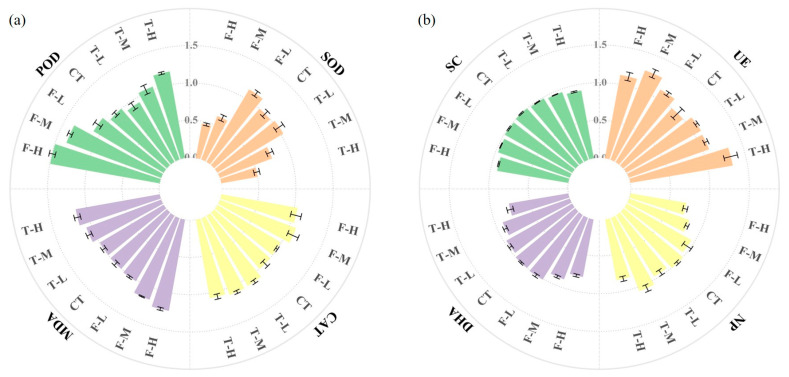
Biochemical responses of *V. natans* roots and rhizosphere sediment to trifluenfuronate and fluopyram exposure. (**a**) Root antioxidant enzymes and MDA. (**b**) Rhizosphere sediment enzymes. Different colors represent different biochemical indicators, and horizontal bars indicate mean ± SD (*n* = 3). Data are presented as fold changes relative to CT (CT = 1.0). Original units before normalization: POD, U⋅mgFW^−1^, SOD and CAT, U⋅gFW^−1^, MDA, nmol·g FW^−1^, SC, mg·d^−1^·g^−1^, UE and DHA, μg·d^−1^·g^−1^, NP, and nmol·h^−1^·g^−1^.

Root oxidative-response indicators were stronger under fluopyram than trifluenfuronate (Figure 2a). In roots, SOD and CAT were significantly increased in T-H, F-M, and F-H compared with CT (*p* < 0.05), whereas MDA showed no significant treatment-related change (*p* > 0.05). POD was lower under T-M and T-H than under CT (*p* < 0.05). Under fluopyram exposure, POD increased at F-L but declined at F-M and F-H. In contrast, MDA did not differ significantly among treatments (*p* > 0.05). In rhizosphere sediment, NP, DHA, and UE generally decreased under pesticide exposure, with the strongest DHA inhibition in F-H. In contrast, SC increased by 31.8% in T-H, and also increased at F-L and F-M but not at F-H.

Together, these results indicate that high-dose fluopyram produced the strongest host–rhizosphere disturbance context for interpreting the microbial community and metagenomic annotation patterns described below.

### 3.2. Responses of Rhizosphere Sediment Microbial Communities

**Figure 3:**
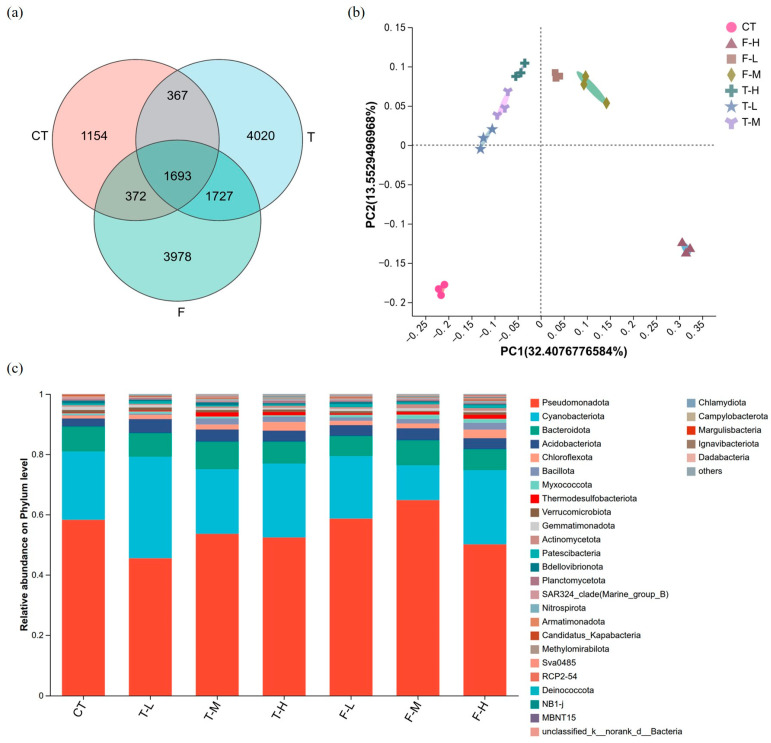
Rhizosphere sediment microbial community responses to trifluenfuronate and fluopyram exposure. (**a**) Venn diagram illustrating shared and unique ASVs among CT, trifluenfuronate (T; pooled across concentrations), and fluopyram (F; pooled across concentrations) groups. (**b**) PCoA ordination based on Bray–Curtis distances. Global PERMANOVA indicated an overall among-group difference (*R*^2^ = 0.71, *p* = 0.001), whereas no individual pairwise comparison remained significant after Benjamini–Hochberg correction. (**c**) Microbial community composition at the phylum level; “others” represents phyla with relative abundance < 1%.

As a descriptive pooled overview, Venn analysis showed that CT, trifluenfuronate-treated groups, and fluopyram-treated groups shared 1693 ASVs (Figure 3a). Both pesticide-exposed sets contained more unique ASVs than CT, with 4020 unique to T and 3978 unique to F, compared with 1154 in CT.

PCoA based on Bray–Curtis distances showed an ordination-level treatment-associated pattern, with PC1 and PC2 explaining 32.41% and 13.55% of the total variance, respectively (Figure 3b). PERMANOVA indicated an overall among-group difference (*R*^2^ = 0.71, *p* = 0.001). The homogeneity test for multivariate dispersion was not significant (PERMDISP, *F* = 0.9548, *p* = 0.485), suggesting that the global PERMANOVA result was not primarily driven by heterogeneity of within-group dispersion. Pairwise comparisons showed relatively large effect sizes for several comparisons involving CT and pesticide-treated groups, although no individual comparison remained significant after Benjamini–Hochberg correction (Table S5). Therefore, the PCoA result was interpreted as an overall ordination-level pattern, rather than statistically resolved pairwise separation among specific treatments. Visually, F-H tended to be more distant from CT than the other fluopyram treatments.

At the phylum level, Pseudomonadota dominated all groups, followed by Cyanobacteriota and Bacteroidota (Figure 3c). Pseudomonadota increased in F-M but decreased in F-H. Cyanobacteriota was more abundant in the T groups, especially T-L.

**Figure 4:**
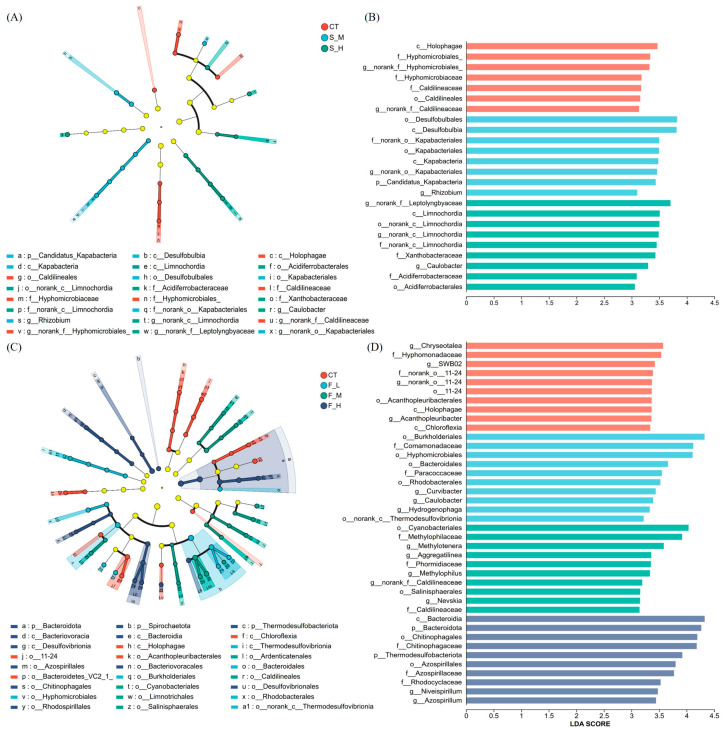
Exploratory LEfSe-screened taxa under trifluenfuronate and fluopyram exposure (LDA > 3.0). (**A**) Cladogram for trifluenfuronate exposure. (**B**) LDA score distribution for trifluenfuronate exposure. (**C**) Cladogram for fluopyram exposure. (**D**) LDA score distribution for fluopyram exposure. In the cladograms, colored nodes indicate taxa significantly enriched in the corresponding groups, whereas light-yellow nodes indicate taxa without significant group differences or without significant contribution to group separation. No genus remained significant after Benjamini–Hochberg correction at *q* < 0.10; therefore, these taxa were not interpreted as FDR-supported biomarkers.

Exploratory LEfSe screening provided hypothesis-generating taxonomic information (Figure 4). In the trifluenfuronate series, LEfSe mainly highlighted features associated with T-M and T-H, including Desulfobulbia/Desulfobulbales in T-M and several Limnochordia-, Leptolyngbyaceae-, Acidiferrobacterales-, Xanthobacteraceae-, and *Caulobacter*-related features in T-H. In the fluopyram series, uncorrected LEfSe highlighted Burkholderiales/Comamonadaceae- and Hyphomicrobiales-related features in F-L, and Bacteroidota-related features, including Bacteroidia and Chitinophagales/Chitinophagaceae, in F-H. However, no genus remained significant after Benjamini–Hochberg correction at *q* < 0.10; these taxa were interpreted as exploratory discriminative features.

### 3.3. Responses of Annotation-Based Metagenomic Profiles

**Figure 5:**
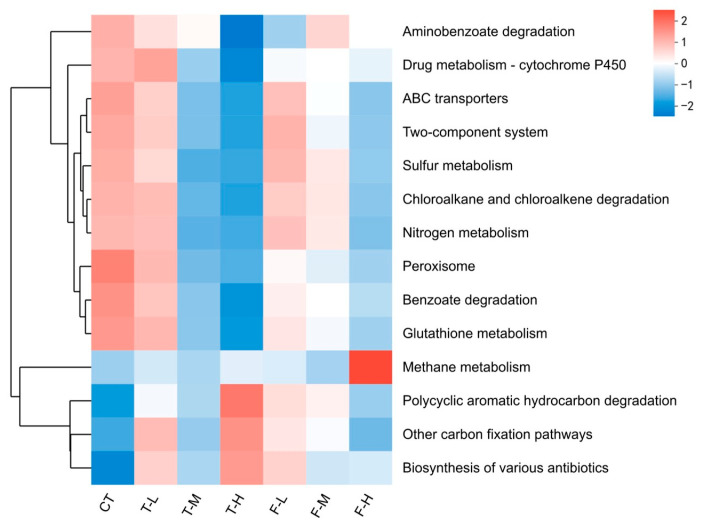
Heatmap of metagenomic KEGG annotation profiles across treatments. Colors indicate scaled relative enrichment or depletion among groups.

The KEGG heatmap provided a descriptive overview of relative metagenomic annotation profiles (Figure 5). Several degradation-related and regulation-related annotation signals were lower under stronger exposure, including aminobenzoate degradation, benzoate degradation, glutathione metabolism, the two-component system, and ABC transporters. T-H showed relative enrichment of annotations grouped as “biosynthesis of various antibiotics”, and a PAH-degradation annotation signal, whereas F-H showed a methane-metabolism-related signal. Nitrogen and sulfur metabolism varied by compound and concentration, rather than following a single monotonic pattern. These results were interpreted as annotation-profile differences, not as direct evidence of pathway activity.

### 3.4. Associations Between Microbial Patterns, Annotation Profiles, and Contextual Variables

**Figure 6:**
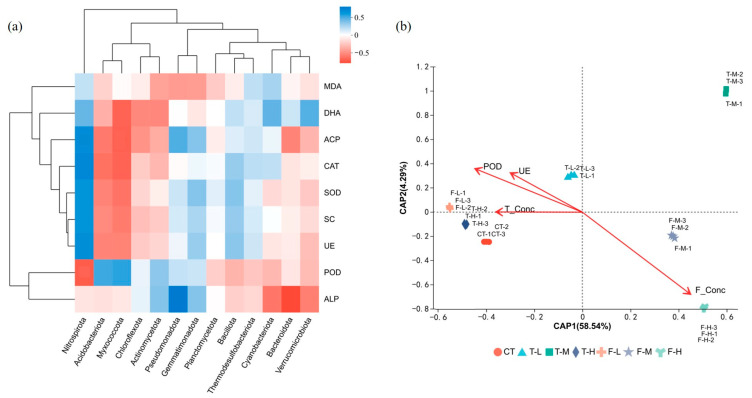
Exploratory correlation and ordination analyses of microbial patterns and environmental variables. (**a**) Spearman correlation heatmap between dominant bacterial phyla and environmental factors. Correlations are shown as exploratory association patterns and were not interpreted as multiple-comparison-corrected biological relationships. (**b**) db-RDA ordination of functional profiles constrained by pesticide concentrations and selected indicators, red arrows indicate explanatory variables, with arrow direction showing their association with the ordination pattern.

The Spearman heatmap was used as an exploratory visualization of phylum-level co-variation patterns with the measured variables (Figure 6a). Several apparent association patterns were observed, including co-variation in Nitrospirota with NP, CAT, SOD, SC, and UE, as well as contrasting patterns for Myxococcota, Pseudomonadota, and Bacteroidota. These patterns were interpreted only as exploratory associations, because multiple-comparison-corrected significance was not used for confirmatory inference. db-RDA further showed that nominal pesticide concentrations were associated with annotation-profile variation (Figure 6b), with CAP1 and CAP2 explaining 58.54% and 4.29% of the constrained variation, respectively. The fluopyram gradient aligned most closely with the higher-dose fluopyram treatments, especially F-H, whereas CT and low-dose treatments clustered near the origin.

## 4. Discussion

Under the tested mass-based exposure conditions, fluopyram was associated with clearer response patterns than trifluenfuronate in the *V. natans*–rhizosphere sediment microcosm. This contrast was observed in rhizosphere bacterial community composition patterns and metagenomic annotation profiles, together with plant growth and sediment-enzyme responses that provided host–sediment context.

### 4.1. Plant and Sediment Enzyme Responses as Host–Sediment Context

Fluopyram caused stronger inhibition of *V. natans* growth and chlorophyll content than trifluenfuronate, particularly at the high dose. This pattern is consistent with the SDHI mode of action of fluopyram, which can affect succinate dehydrogenase-linked respiratory energy metabolism, although the present data do not directly measure mitochondrial activity [19]. The concurrent pigment reduction and induction of SOD and CAT suggest a greater physiological burden under fluopyram exposure in this macrophyte. However, unchanged MDA should be interpreted only as no detectable increase in MDA-based lipid peroxidation under the tested conditions; MDA alone cannot exclude other oxidative or metabolic responses [20]. In comparison, trifluenfuronate caused weaker growth inhibition and a more limited antioxidant response pattern over the same exposure period.

Rhizosphere sediment enzymes also showed treatment-associated changes. Declines in UE, DHA, and NP suggest lower sediment enzyme activities related to nutrient transformation, redox-related microbial activity, and phosphorus mineralization, respectively, with the strongest overall inhibition generally observed under high-dose fluopyram [21,22]. However, extracellular enzyme activities are influenced by microbial abundance and activity, substrate availability, root-derived inputs, local physicochemical conditions, and sediment matrix effects [23]. Thus, these enzyme responses should be interpreted as integrated rhizosphere functional indicators, rather than direct evidence for changes in a single microbial process. By contrast, T-H increased sucrase activity, indicating a selective treatment-associated response in rhizosphere carbon-processing potential [24]. Because extracellular enzyme activities can be influenced by microbial activity, substrate availability, root-derived inputs, and sediment matrix effects, this SC response should be interpreted as an integrated rhizosphere indicator, rather than evidence for a single underlying process [25]. Overall, the plant and enzyme results provided host–sediment context for interpreting the microorganism-centered results described below.

### 4.2. Microbial Community and Annotation-Based Functional Patterns

Microbial community analyses were broadly consistent with the plant and enzyme responses. Accordingly, the 16S rRNA gene results are discussed as ordination-level community composition patterns and exploratory taxonomic signals, rather than as confirmed treatment-specific biomarkers [15,16,17]. LEfSe-screened taxa were therefore used only as hypothesis-generating features, because they were not supported as significant genera after multiple-testing correction. Within this framework, fluopyram showed a clearer ordination-level response pattern, especially at the high dose, which coincided with the strongest plant growth inhibition and enzyme suppression observed under the same treatment [22]. By comparison, trifluenfuronate produced a milder taxonomic composition pattern, consistent with its more selective response pattern [7]. These results support the view that pesticide exposure can restructure rhizosphere bacterial communities, but they do not establish direct causal links between specific taxa and plant or enzyme responses [1].

The metagenomic results provided an additional annotation-based view of these response patterns [26]. Several xenobiotic-, transport-, and regulation-related annotation signals decreased under stronger exposure, while F-H showed a methane-metabolism-related signal and T-H showed relative enrichment of secondary-metabolism-related annotations [1]. Because transcriptional evidence, pathway-specific enzyme assays, and pesticide degradation measurements were not included, these metagenomic outputs should be interpreted as relative KEGG annotation profiles [27]. Thus, the 16S and shotgun metagenomic datasets were integrated at the response-pattern level: 16S data described community composition patterns, whereas metagenomic data described relative functional annotation profiles. In this integrated interpretation, fluopyram, especially at the high dose, showed clearer ordination-level community composition patterns and broader relative annotation-profile differences, whereas trifluenfuronate showed milder community composition patterns and more selective annotation changes [7,19].

### 4.3. Integrated Interpretation

Taken together, the results suggest two contrasting response patterns in the *V. natans*–rhizosphere sediment system. Fluopyram was associated with a stronger multi-endpoint response pattern, especially at the high dose, including clearer ordination-level microbial community composition patterns and broader relative metagenomic annotation-profile differences, accompanied by stronger plant-growth inhibition and greater suppression of several rhizosphere sediment enzyme activities. Trifluenfuronate was associated with a milder and more selective pattern, with relatively stable plant growth but detectable shifts in antioxidant regulation, SC activity, microbial composition, and secondary-metabolism-related annotations. These results indicate that rhizosphere microbial community and metagenomic annotation endpoints, interpreted together with plant and sediment-enzyme context, can provide a microorganism-centered framework for comparing pesticide responses in sediment-associated freshwater microcosms.

These patterns should be interpreted under the tested 28-day, single-pulse *V. natans* microcosm conditions. Initial pesticide measurements confirmed the starting exposure levels after single-pulse dosing, but temporal dissipation, sediment residues, bioavailable pesticide fractions, and full water–sediment–plant partitioning were not determined. Therefore, responses observed at 1 mg L^−1^ should be viewed as high-end runoff/stress-scenario responses rather than routine field-level responses. In addition, the use of three independent microcosms per treatment limited the statistical power of omics-based and multivariate analyses; therefore, microbial and metagenomic results were interpreted as exploratory and complementary response-pattern evidence. Future studies incorporating residue time courses, bioavailable exposure metrics, higher replication and recovery-phase observations would help to evaluate the persistence and field relevance of these response patterns.

## 5. Conclusions

Under the tested microcosm conditions, fluopyram was associated with clearer rhizosphere microorganism-associated responses than trifluenfuronate, as indicated by ordination-level microbial community patterns and relative metagenomic annotation-profile differences. These patterns were accompanied by stronger plant-growth inhibition and greater suppression of several rhizosphere sediment enzyme activities under high-dose fluopyram exposure. Overall, this study supports the use of rhizosphere microbial community and metagenomic annotation endpoints, interpreted together with plant and sediment-enzyme context, for comparing pesticide responses in sediment-associated freshwater microcosms. Future studies with higher replication, exposure time courses, and recovery observations are needed to assess the persistence and ecological relevance of these short-term patterns.

## Data Availability

The raw 16S rRNA gene sequencing data have been deposited in the NCBI database under accession number PRJNA1422131. Other data supporting the findings of this study are available from the corresponding author upon reasonable request.

## Supplementary Materials

The following supporting information can be downloaded at: https://www.mdpi.com/article/10.3390/microorganisms14051166/s1, Table S1: Physicochemical properties of the lake water and sediment samples; Text S1: Runoff-scenario calculation for the high nominal exposure level [5,28,29,30,31,32,33,34,35]; Table S2: Verification of measured-to-nominal pesticide concentrations and calibration-range coverage in overlying water immediately after dosing; Text S2: Analytical method for pesticide quantification; Table S3: Mobile phase and MS/MS ion transitions used for pesticide quantification; Text S3: Verification of the preconcentration procedure; Table S4A: Calibration linearity and matrix-effect assessment for LC-MS/MS quantification; Table S4B: S/N-based estimated LODs and LOQs using low-concentration lake-water samples after 20-fold preconcentration; Text S4: Sequencing Data Quality and Taxonomic Summary; Figure S1: Rarefaction curves of bacterial community Shannon diversity indices in rhizosphere sediment samples after normalization; Table S5: Pairwise PERMANOVA comparisons of rhizosphere sediment bacterial community composition based on Bray-Curtis distances; Text S5: Supplementary Methods for Microcosm Preparation and Contextual Plant–Sediment Measurements.
