# Supplementary material for: Rhizosphere Microbial Community and Metagenomic Annotation Responses in a Vallisneria natans–Sediment Microcosm Exposed to Trifluenfuronate and Fluopyram
Source: Microorganisms. 2026 May 21;14(5):1166. doi: 10.3390/microorganisms14051166 (PMC13210105; doi:10.3390/microorganisms14051166)
Supplement: Supplementary file 1 [file microorganisms-14-01166-s001.zip › microorganisms-4285927-supplementary.pdf]

Supplementary Materials:

Table S1. Physicochemical properties of the lake water and sediment samples.

Medium	Parameter	Unit	Value (Mean ± SD, *n*=3)
Lake Water (Collected from Rihu Lake)	pH	/	6.96 ± 0.3
	Dissolved Oxygen (DO)	$mg\cdot L^{-1}$	7.16 ± 0.21
	Temperature	℃	24.0 ± 1
	Conductivity	$\mu S\cdot cm^{-1}$	578 ± 11
Sediment (Collected from Rihu Lake)	pH	/	7.67 ± 0.04
	Available Nitrogen (AN)	$mg\cdot kg^{-1}$	147.58 ± 5.23
	Available Phosphorus (AP)	$mg\cdot kg^{-1}$	76.57 ± 3.86
	Organic Matter (OM)	$g\cdot kg^{-1}$	18.58 ± 0.35

Text S1. Runoff-scenario calculation for the high nominal exposure level

The high nominal fluopyram level used in this study was not intended to represent a routine concentration in most natural freshwater systems. Instead, it was included as a conservative near-field agricultural ditch/runoff scenario. We used a simple mass-balance estimation, based on currently reported fluopyram application rates and plausible edge-of-field runoff assumptions.

Published regulatory and label-related information indicates that fluopyram may be applied at approximately 150-300 g active ingredient (a.i.) ha^{-1} per application for some crop uses, while annual maximum use levels can approach approximately 500 g a.i. ha^{-1} depending on formulation and use pattern[1–3]. Therefore, a representative application range of 150-300 g a.i. ha^{-1} per application, together with a conservative upper seasonal/annual level of 500 g a.i. ha^{-1}, was used for the scenario calculation.

The potential concentration of fluopyram in a small agricultural ditch receiving runoff from an adjacent treated field was estimated using the following mass-balance equation:

$$\mathrm{C_{ditch}} = \frac{D \times \mathrm{f_{runoff}}}{\mathrm{V_{ditch}}}$$

where $\mathrm{C_{ditch}}$ is the estimated fluopyram concentration in ditch water ($mg\cdot L^{-1}$), D is the applied fluopyram mass per unit field area (g a.i. ha^{-1}), $\mathrm{f_{runoff}}$ is the fraction of the applied active ingredient entering the ditch during a runoff event, and $\mathrm{V_{ditch}}$ is the ditch water volume receiving runoff from 1 ha of treated field ($m^3\cdot ha^{-1}$). Because 1 $g\cdot m^{-3}$ is equivalent to 1 $mg\cdot L^{-1}$, the equation directly yields concentrations in $mg\cdot L^{-1}$ when D is expressed in $g\cdot ha^{-1}$ and $\mathrm{V_{ditch}}$ in $m^3\cdot ha^{-1}$.

For a near-field agricultural ditch scenario, a 1-ha treated field draining into an adjacent shallow ditch of approximately 100 m length and 0.1-0.2 m^2 effective water cross-sectional area would correspond to a ditch water volume of approximately 10-20 m^3 ha^{-1}. Under this small-volume receiving-water scenario, an application rate of 300 g a.i. ha^{-1} combined with 3.3% transfer of the applied mass into a 10 m^3 ditch would yield an estimated concentration of approximately 0.99 $mg\cdot L^{-1}$:

$$\mathrm{C_{ditch}} = \frac{300 \times 0.033}{10} = 0.99 mg \cdot L^{-1}$$

Similarly, an application rate of 500 g a.i. ha^{-1} with 2% transfer into a 10 m^3 ditch, or 4% transfer

into a 20 m^3 ditch, would also yield an estimated concentration close to 1 mg·L^{-1}. These transfer fractions are within the broad range reported for pesticide edge-of-field losses under runoff conditions, particularly when rainfall or irrigation runoff occurs shortly after pesticide application[4]. Similar scenario-based drainage-ditch studies have used pesticide concentrations in the sub-mg to mg·L^{-1} range to simulate near-field runoff inputs based on recommended application rates and assumed runoff fractions[5]. Therefore, the 1 mg·L^{-1} treatment was considered a conservative high-end nominal level for comparing system-level responses under stronger near-field chemical pressure.

This scenario-based rationale is consistent with the use of conservative surface-water exposure scenarios in pesticide aquatic risk assessment. The FOCUS surface-water guidance describes standard scenarios for estimating predicted environmental concentrations in surface water and sediment, including pesticide inputs via runoff, drainage, and spray drift[6]. Recent studies also support the importance of runoff-driven pesticide transport. Runoff and erosion from agricultural fields after rainfall have been identified as major pesticide transport processes into surface waters. Rainfall intensity, rainfall amount, and the interval between pesticide application and rainfall strongly influence pesticide losses in runoff, and major losses can occur when substantial rainfall occurs during or shortly after pesticide application[7]. High-frequency monitoring studies further show that small agricultural catchments can experience short-lived pesticide concentration peaks that may be missed by conventional low-frequency sampling[8]. Agricultural storm-drainage inlets have also been shown to strengthen the hydrological connectivity between surface runoff and receiving waters. In addition, recent runoff-treatment studies have used controlled pesticide inputs to simulate agricultural runoff entering vegetated ditch or treatment systems[9]. Therefore, the 1 mg·L^{-1} treatment was retained as a conservative high-end single-pulse runoff/stress scenario for comparative response assessment, not as evidence of a routinely monitored environmental concentration.

Because compound-specific field monitoring data for trifluenfuronate remain limited, the same nominal concentration gradient was used to enable a controlled mass-based comparison between the two pesticides.

Table S2. Verification of measured-to-nominal pesticide concentrations and calibration-range coverage in overlying water immediately after dosing.

Treatment group	Nominal concentration (mg·L^{-1})	Pretreatment before LC-MS/MS	Theoretical concentration in final analytical solution (ng·mL^{-1})	Matrix-matched calibration range (ng·mL^{-1})	Measured initial concentration (mg·L^{-1})	Measured-to-nominal (%)	RSD (%)
CT	0	Direct analysis	—	—	ND	—	—
T-L	0.01	20-fold preconcentration	200	65.85 – 3292.65	0.0088 ± 0.0003	88	3.14

T-M	0.1	Direct analysis	100	65.85 – 3292.65	0.094 ± 0.012	94	12.8
T-H	1	Direct analysis	1000	65.85 – 3292.65	0.98 ± 0.011	98	1.1
F-L	0.01	20-fold preconcentration	200	11.43 – 571.34	0.0089 ± 0.0003	89	3.68
F-M	0.1	Direct analysis	100	11.43 – 571.34	0.097 ± 0.008	97	8.2
F-H	1	20-fold dilution	50	11.43 – 571.34	0.99 ± 0.01	99	1

Notes: Values are presented as mean ± SD (n = 3). ND, not detected. The theoretical concentration in the final analytical solution was calculated after considering the corresponding preconcentration or dilution factor before LC-MS/MS analysis. For pesticide-treated groups, all final analytical concentrations were within the corresponding matrix-matched calibration ranges. The measured-to-nominal ratio was calculated as the measured initial concentration divided by the nominal concentration. These results were used to verify that the measured exposure concentrations were close to the nominal concentrations and that the final analytical concentrations fell within the corresponding matrix-matched calibration ranges. The measured-to-nominal ratios of 88–99% and RSDs of 1.0–12.8% were considered acceptable for verifying exposure concentrations in this fit-for-purpose analysis.

Text S2. Analytical method for pesticide quantification

Analytical standards of trifluenfuronate and fluopyram with purities of 98.5% and 96%, respectively, were used. For LC-MS/MS calibration, matrix-matched calibration, and analytical validation, standard stock solutions were prepared separately in LC-grade acetonitrile at 100 mg L^{-1}. These acetonitrile-based solutions were used only for analytical calibration and validation, whereas the microcosm exposure system used acetone-based dosing solutions as described in Sections 2.1 and 2.2 of the main text. To validate quantification near the low-dose level, low-level matrix-spiked lake-water samples were prepared at 0.010 mg L^{-1} using acetonitrile-based analytical working solutions. The final acetonitrile content in these analytical validation samples before pretreatment was approximately 1.0% (v/v), and solvent-matched validation blanks were processed in parallel. This acetonitrile content refers only to the analytical validation samples and not to the *V. natans*–sediment microcosm exposure system. After spiking, the samples were vortex-mixed and shaken at 180 rpm for 30 min to ensure sufficient mixing and equilibration. The pH of the water samples was not adjusted before pretreatment.

For the low-dose treatments, 20.0 mL of overlying-water sample was collected and subjected to 20-fold preconcentration before LC-MS/MS analysis. The samples were centrifuged at 4000 rpm for 20 min to remove suspended particles. The supernatant was loaded onto a Waters Oasis HLB SPE cartridge, 1 cc / 30 mg / 30 μm. Before sample loading, the SPE cartridge was conditioned sequentially with 5 mL of methanol and 5 mL of ultrapure water. The water sample was loaded onto the cartridge at a flow rate of approximately 1–2 mL·min⁻

[1]. After sample loading, the cartridge was rinsed with 5 mL of ultrapure water and dried under vacuum for 15–30 min. The target pesticide was eluted with 5 mL of methanol. The eluate was collected and gently evaporated under a nitrogen stream at 35–40 ℃ to near dryness. The residue was reconstituted with the initial LC-MS/MS mobile phase or methanol/water solution and adjusted to a final volume of 1.00 mL. The final extract was vortex-mixed, filtered through a 0.22 μm organic membrane filter, and transferred to an LC-MS/MS vial for instrumental analysis. The theoretical enrichment factor was 20, calculated from the ratio of the initial sample volume, 20.0 mL, to the final reconstitution volume, 1.00 mL. Therefore, the theoretical concentration of the target pesticide in the final analytical solution was approximately 0.20 mg·L^{-1} for the 0.01 mg·L^{-1} low-dose treatment, assuming complete extraction recovery and no analyte loss during pretreatment.

Initial concentrations of trifluenfuronate and fluopyram in the overlying water were determined using high-performance liquid chromatography coupled with tandem mass spectrometry (HPLC-MS/MS). The analytical system consisted of a Thermo Fisher Ultimate 3000 HPLC system coupled to a TSQ Quantis triple-quadrupole mass spectrometer equipped with an electrospray ionization source (Thermo Fisher Scientific, USA). Chromatographic separation was performed using a Thermo Fisher Hypersil GOLD C18 column (2.1 × 150 mm, 3 μm).

The analysis was conducted in positive electrospray ionization mode (ESI^+) using multiple reaction monitoring (MRM). For trifluenfuronate, the mobile phase consisted of methanol and 0.1% formic acid aqueous solution containing 5 mmol L^{-1} ammonium acetate. The precursor ion was m/z 345.2, and the monitored product ions were m/z 145.1 and 115.1, with m/z 145.1 used as the quantifier ion and m/z 115.1 used as the qualifier ion. For fluopyram, the mobile phase consisted of 0.1% formic acid in acetonitrile and 0.1% formic acid in water. The precursor ion was m/z 397.04, and the monitored product ions were m/z 207.97 and 144.88, with m/z 207.97 used as the quantifier ion and m/z 144.88 used as the qualifier ion.

Because the low-dose treatments were near the low end of the calibration range, overlying-water samples from the low-dose treatments were reanalyzed after 20-fold preconcentration. Quantification was performed using matrix-matched external calibration. The matrix-matched calibration range was 11.43–571.34 ng/mL for fluopyram and 65.85–3292.65 ng/mL for trifluenfuronate, with R^2 values of 0.996 and 0.997, respectively. The lowest matrix-matched calibration levels were 11.43 ng·mL^{-1} for fluopyram and 65.85 ng·mL^{-1} for trifluenfuronate, corresponding to sample-equivalent concentrations of 0.00057 mg·L^{-1} and 0.00329 mg·L^{-1} in the original water samples, respectively, after 20-fold preconcentration. To validate quantification near the low-dose level, matrix-spiked overlying-water samples were prepared at 0.010 mg·L^{-1} and processed using the same 20-fold preconcentration procedure. The back-calculated concentrations were 0.0090 ± 0.0002 mg·L^{-1} for trifluenfuronate and 0.0090 ± 0.0002 mg·L^{-1} for fluopyram, corresponding to recoveries of 89.8 ± 1.8% and 90.0 ± 2.3%, respectively. Repeatability of the preconcentration validation was acceptable, with relative standard deviations of 1.9% for trifluenfuronate and 2.5% for fluopyram. Matrix effects were evaluated by comparing solvent-based and matrix-matched calibration slopes (Table S4A), and matrix-matched calibration was used for quantification. The matrix-spiked recovery results further supported the reliability of initial low-dose exposure verification after preconcentration.

Table S3. Mobile phase and MS/MS ion transitions used for pesticide quantification.

Compound	Mobile phase	Ion source	Precursor ion (m/z)	Product ions (m/z)
Trifluenfuronate	Methanol: 0.1% formic acid in water + 5 mmol L^{-1} ammonium acetate	ESI+	345.2	*145.1/115.1
Fluopyram	0.1% formic acid in acetonitrile: 0.1% formic acid in water	ESI+	397.04	*207.97/144.88

Asterisks indicate quantifier ions; the other product ions were used as qualifier ions.

Text S3. Verification of the preconcentration procedure

For the low-dose treatments, overlying-water samples were concentrated 20-fold before instrumental analysis. Matrix-matched external calibration was performed using the compound-specific calibration ranges shown in Table S4A, with R^2 values > 0.99 for both compounds. The lowest calibration level was 0.05 mg·L^{-1} in the concentrated solution, corresponding to a sample-equivalent quantification level of 0.0025 mg·L^{-1} in the original water sample after 20-fold preconcentration. To evaluate the reliability of the preconcentration procedure, three independently processed matrix-spiked water samples were prepared at 0.010 mg·L^{-1} and subjected to the same 20-fold preconcentration procedure. For trifluenfuronate, the measured concentrations in the concentrated solutions were 0.180, 0.176, and 0.183 mg·L^{-1}, corresponding to a mean concentrated-solution concentration of 0.180 ± 0.004 mg·L^{-1} and a back-calculated original-water concentration of 0.0090 ± 0.0002 mg·L^{-1}. The corresponding recovery was 89.8 ± 1.8%. For fluopyram, the measured concentrations in the concentrated solutions were 0.184, 0.175, and 0.181 mg·L^{-1}, corresponding to a mean concentrated-solution concentration of 0.180 ± 0.005 mg·L^{-1} and a back-calculated original-water concentration of 0.0090 ± 0.0002 mg·L^{-1}. The corresponding recovery was 90.0 ± 2.3%.

These results indicate that the low-dose concentrations reported in Table S2 were above the sample-equivalent quantification level defined by the lowest calibration level and could be quantified after 20-fold preconcentration.

Table S4A. Calibration linearity and matrix-effect assessment for LC-MS/MS quantification.

**Compound**	**Calibration type**	**Regression equation**	**R²**	**Calibration range (ng·mL^{-1})**	**Residual range (%)**	**Matrix effect (%)**
Fluopyram	Solvent-based	Y=583.8756X + 156.4588	0.999	11.43-571.34	-15.58 to 2.15	-
	Matrix-matched	Y=504.9355X + 171.4622	0.996		-14.37 to 5.09	-13.52
Trifluenfuronate	Solvent-based	Y=15.854X + 526.074	0.999	65.85-3292.65	-0.53 to 0.12	-

Matrix-matched	Y=13.154X + 631.235	0.997	-13.20 to 3.80	-17.03

Notes: X represents the concentration in the final analytical solution (ng·mL^{-1}), and Y represents the peak area. Residuals represent the relative deviation between back-calculated and nominal calibration concentrations. Matrix effect was calculated as:

$$Matrix\ effect(\%) = \left[\frac{slope\ of\ matrix - matched\ calibration\ curve}{slope\ of\ solvent - based\ calibration\ curve} - 1\right] \times 100$$

Table S4B. S/N-based estimated LODs and LOQs using low-concentration lake-water samples after 20-fold preconcentration.

Compound	Low-level sample used for estimation (mg·L^{-1})	Pretreatment	Back-calculated concentrations in final analytical solution (ng·mL^{-1}, n = 3)	S/N values, n = 3	Mean S/N ± SD	Mean estimated LOD in final solution, S/N = 3 (ng·mL^{-1})	Mean estimated LOQ in final solution, S/N = 10 (ng·mL^{-1})	Method-estimated LOD in original water (mg·L^{-1})	Method-estimated LOQ in original water (mg·L^{-1})
Fluopyram	0.01	20-fold preconcentration	177.00, 172.00, 185.00	631, 584, 571	595 ± 32	0.9	3	4.50×10^{-5}	1.50×10^{-4}
Trifluenfuronate	0.01	20-fold preconcentration	176.00, 181.00, 170.00	247, 189, 163	200 ± 43	2.71	9.04	1.36×10^{-4}	4.52×10^{-4}

Notes: Blank lake-water samples showed no detectable target peaks at the retention times of fluopyram or trifluenfuronate. Signal-to-noise ratios were obtained from replicate low-concentration lake-water samples processed using the same 20-fold preconcentration procedure. Because the S/N values of the low-concentration samples were substantially higher than 10, the LOD and LOQ values should be regarded as S/N-based method sensitivity estimates rather than formal experimentally confirmed detection and quantification limits. LODs and LOQs in the final analytical solution were estimated according to:

$$C_{LOD} = C \times \frac{3}{S/N}$$

$$C_{LOQ} = C \times \frac{10}{S/N}$$

where C is the back-calculated concentration in the final analytical solution. The final reported LOD and LOQ values were calculated as the mean of the three replicate estimates. Method-estimated LODs and LOQs in the original water samples were obtained by dividing the estimated concentrations in the final analytical solution by the 20-fold preconcentration factor and converting ng·mL^{-1} to mg·L^{-1}. These S/N-based LOD and LOQ values represent

method sensitivity estimates for the 20-fold preconcentration–LC-MS/MS procedure and were not used to extend the established matrix-matched calibration ranges. Quantification of exposure samples was performed only when the final analytical concentrations were within the corresponding matrix-matched calibration ranges.

Text S4. Sequencing Data Quality and Taxonomic Summary

High-throughput sequencing of the 16S rRNA gene was processed through the Majorbio Cloud Platform. After quality control and normalization, all libraries used for downstream community analysis were rarefied to 28,669 valid sequences per sample, allowing standardized between-group comparison of microbial diversity and community composition. The ASV numbers in individual samples were approximately 10^3 per sample. The finalized species-statistics output identified 13,312 ASVs across the 21 rhizosphere sediment samples, encompassing 61 phyla, 169 classes, 377 orders, 592 families, 1,119 genera, and 2,267 species. Because the rarefaction curves approached saturation across samples, the sequencing depth was considered adequate for the between-group diversity and community-composition comparisons performed in this study.

Rarefaction curves based on the Shannon diversity index were constructed to evaluate the adequacy of sequencing depth. As illustrated in Fig. S1, the curves approached a plateau and became nearly parallel to the x-axis when sequencing depth reached approximately 20,000 reads. These results support adequate sequencing depth for the between-group diversity and community-composition comparisons performed in this study.

Because the downstream 16S analyses were focused on diversity and overall community-composition patterns, rarefaction to an even sequencing depth was used for between-sample standardization. Alternative count-based or compositional differential-abundance approaches, such as DESeq2 or CLR transformation, were not used for confirmatory taxon-level inference because no differential taxon was used as a central conclusion of this study.

Figure S1. Rarefaction curves of bacterial community Shannon diversity indices in rhizosphere sediment samples after normalization.

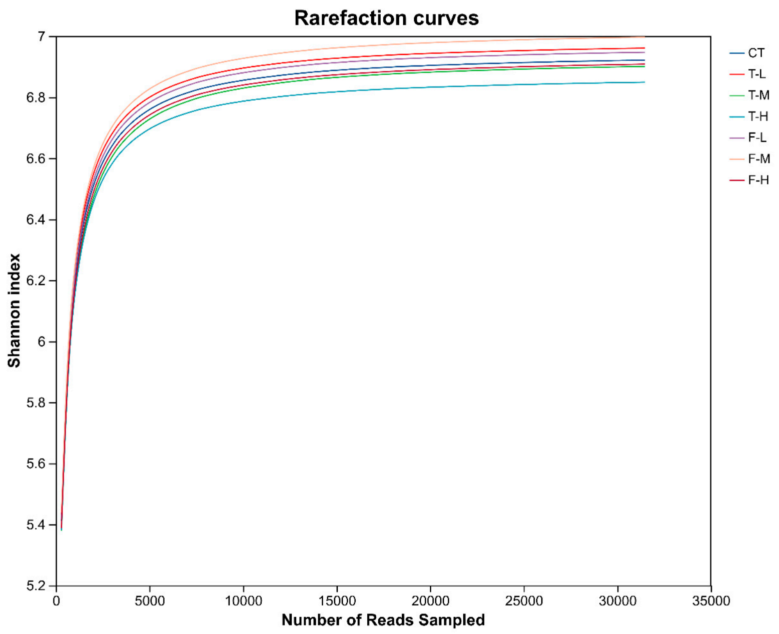

Table S5. Pairwise PERMANOVA comparisons of rhizosphere sediment bacterial community composition based on Bray-Curtis distances.

Group 1	Group 2	R^2	*F* value	*p* value	BH-adjusted *p* value
CT	T-L	0.6216	6.5718	0.100	0.100
CT	T-M	0.6344	6.9404	0.100	0.100
CT	T-H	0.7078	9.6905	0.100	0.100
CT	F-L	0.7135	9.9631	0.100	0.100
CT	F-M	0.7132	9.9468	0.100	0.100
CT	F-H	0.7310	10.8692	0.100	0.100
T-L	T-M	0.3741	2.3904	0.100	0.100
T-L	T-H	0.4661	3.4915	0.100	0.100
T-L	F-L	0.5324	4.5550	0.100	0.100
T-L	F-M	0.5732	5.3720	0.100	0.100
T-L	F-H	0.6359	6.9874	0.100	0.100
T-M	T-H	0.4572	3.3686	0.100	0.100
T-M	F-L	0.4604	3.4126	0.100	0.100
T-M	F-M	0.5013	4.0206	0.100	0.100
T-M	F-H	0.6055	6.1388	0.100	0.100
T-H	F-L	0.5159	4.2622	0.100	0.100
T-H	F-M	0.5195	4.3246	0.100	0.100
T-H	F-H	0.6070	6.1784	0.100	0.100
F-L	F-M	0.3952	2.6142	0.100	0.100
F-L	F-H	0.5510	4.9080	0.100	0.100

F-M	F-H	0.4793	3.6818	0.100	0.100

Pairwise PERMANOVA was conducted using three independent biological replicates per treatment. P values were adjusted using the Benjamini-Hochberg procedure. No individual pairwise comparison remained statistically significant after correction. R^2 values are provided as effect-size indicators of treatment-level community separation. Homogeneity of multivariate dispersion was evaluated using PERMDISP based on the same Bray-Curtis distance matrix, and no significant among-group dispersion difference was detected (F = 0.9548, p = 0.485).

Text S5. Supplementary Methods for Microcosm Preparation and Contextual Plant–Sediment Measurements

S5.1. Plant acclimation and material preparation

Vallisneria natans plants were obtained from a horticultural company in Nanjing, China, and cleaned thoroughly with tap water to remove surface debris. Before microcosm construction, plants were pre-cultured for one week under controlled laboratory conditions, with a light intensity of 90 μmol m^{-2} s^{-1}, a 12 h light/12 h dark photoperiod, and a temperature of 24 ± 2 °C.

Surface water and sediment were collected from Rihu Lake, located on the campus of Fudan University, Shanghai, China. The sampling location was 31°20′27″ N, 121°30′46″ E. Sediment samples were gently homogenized and sieved through stainless-steel sieves to remove coarse debris, including gravel and plant residues. Basic physicochemical properties of the collected water and sediment are provided in Table S1.

Analytical standards of trifluenfuronate and fluopyram were purchased from Shandong United Pesticide Industry, Jinan, China, and Nanjing Agrochemical Co., Ltd., Nanjing, China, respectively. The purities of trifluenfuronate and fluopyram were 98.5% and 96%, respectively. Acetone-based stock solutions were prepared and stored at 4 °C in the dark until use. Detailed LC-MS/MS analytical procedures and validation results for exposure verification are provided in Supplementary Texts S2–S3 and Tables S2–S4.

S5.2. Microcosm construction and sampling procedure

Each 5-L cylindrical plexiglass container received 1.5 kg of pre-treated sediment. Three to four *V. natans* individuals without ramets were transplanted into the sediment in each container. The initial fresh weight and mean plant length were 8.02 ± 0.11 g and 9.2 ± 1.7 cm, respectively. Lake water, 4 L per container, was then added slowly along the inner wall of each container to minimize sediment resuspension.

After 28 days of exposure, the overlying water was siphoned off. Plants were carefully removed from the sediment, and loosely attached sediment particles were gently shaken off. Rhizosphere sediment tightly associated with the roots was collected for microbial, metagenomic, and enzyme analyses. Root length, leaf length, and fresh weight were measured for each individual plant. For statistical analysis, measurements from the three to four plants within the same container were averaged to obtain one value per microcosm, thereby avoiding pseudoreplication. Leaf tissue, root tissue, and rhizosphere sediment were collected and stored for subsequent analyses.

S5.3. Plant growth and chlorophyll measurements

Surface water and residual moisture were gently blotted from plant tissues with lint-free paper

before fresh weight was measured.

Chlorophyll a (Chl a) and chlorophyll b (Chl b) were quantified following ethanol extraction. Briefly, 0.10 g of cleaned leaf tissue was immersed in 10 mL of 96% (v/v) ethanol and incubated in the dark for 24 h at room temperature. Absorbance was recorded at 645 and 663 nm using a microplate reader (SpectraMax M2, Molecular Devices, USA). Because the chlorophyll equations are based on 1-cm pathlength absorbance, microplate absorbance values were converted to 1-cm pathlength-equivalent values using a validated correction factor of 1.64. Chl a and Chl b concentrations were calculated based on absorbance at 663 nm (A663) and 645 nm (A645) using the following equations:

$$Chl\ a = 12.7 \times A\_663 - 2.69 \times A\_645$$

$$Chl\ b = 22.9 \times A\_645 - 4.68 \times A\_663$$

S5.4. Root oxidative-response measurements

Fresh root tissue, 0.05 g per sample, was rapidly frozen in liquid nitrogen, ground to a fine powder, and homogenized in 0.1 M phosphate-buffered saline. The homogenate was centrifuged at 10,000 rpm for 10 min at 4 °C, and the supernatant was collected for biochemical analyses.

Malondialdehyde content and antioxidant enzyme activities, including superoxide dismutase, peroxidase, and catalase, were determined using commercial assay kits from Nanjing Jiancheng Bioengineering Institute, China, according to the manufacturer's instructions. These indicators were used as host-context variables to describe plant oxidative-response status in the microcosms.

S5.5. Rhizosphere sediment enzyme measurements

Rhizosphere sediment samples were stored at −20 °C before analysis. Sediment samples were freeze-dried at −55 °C for 48 h, ground, sieved through a 100-mesh screen, and stored at −20 °C until enzyme analysis.

Activities of urease, dehydrogenase, neutral phosphatase, and sucrase were quantified using commercial assay kits from Nanjing Jiancheng Bioengineering Institute, China, according to the manufacturer's instructions. These enzymes were selected as integrated rhizosphere sediment indicators of nitrogen transformation, microbial redox activity, phosphorus mineralization, and labile-carbon turnover, respectively.

References for Supplementary Materials

1. Bayer Crop Science New Zealand Luna® Privilege 250SC Product Label 2024.
2. United States Environmental Protection Agency Pesticide Product Label: Fluopyram 500 SC 2016.
3. Environmental Protection Authority New Zealand *Science Memorandum: APP203261 – Luna Privilege*; Environmental Protection Authority New Zealand: Wellington, New Zealand, 2018;
4. Zhang, X.; Goh, K.S. Evaluation of Three Models for Simulating Pesticide Runoff from Irrigated Agricultural Fields. *J. Environ. Qual.* 2015, *44*, 1809–1820, doi:10.2134/jeq2014.11.0474.
5. Bennett, E.R.; Moore, M.T.; Cooper, C.M.; Smith, S., Jr.; Shields, F.D., Jr.; Drouillard, K.G.; Schulz, R. Vegetated Agricultural Drainage Ditches for the Mitigation of Pyrethroid-

Associated Runoff. *Environ. Toxicol. Chem.* 2005, *24*, 2121–2127, doi:10.1897/04-357R.1.
6. FOCUS *Generic Guidance for FOCUS Surface Water Scenarios*; Version 1.4.; FOCUS Working Group on Surface Water Scenarios, 2015;
7. Chen, C.; Guo, W.; Ngo, H.H. Pesticides in Stormwater Runoff—a Mini Review. *Front. Environ. Sci. Eng.* 2019, *13*, 72, doi:10.1007/s11783-019-1150-3.
8. la Cecilia, D.; Dax, A.; Ehmann, H.; Koster, M.; Singer, H.; Stamm, C. Continuous High-Frequency Pesticide Monitoring to Observe the Unexpected and the Overlooked. *Water Res. X* 2021, *13*, 100125, doi:10.1016/j.wroa.2021.100125.
9. Phillips, B.M.; Cahn, M.; Voorhees, J.P.; McCalla, L.; Siegler, K.; Chambers, D.L.; Lockhart, T.R.; Deng, X.; Tjeerdema, R.S. An Integrated Vegetated Treatment System for Mitigating Imidacloprid and Permethrin in Agricultural Irrigation Runoff. *Toxics* 2021, *9*, 7, doi:10.3390/toxics9010007.
